# The Immediate and Delayed Post-Debridement Effects on Tissue Bacterial Wound Counts of Hypochlorous Acid Versus Saline Irrigation in Chronic Wounds

**Published:** 2016-12-01

**Authors:** John M. Hiebert, Martin C. Robson

**Affiliations:** Truman Medical Center, University of Missouri–Kansas City; and Division of Plastic Surgery, University of South Florida, Tampa

**Keywords:** ultrasonic debridement, bioburden, irrigation, hypochlorous acid, wound closure

## Abstract

**Introduction:** Wound debridement is considered essential in chronic wound management. Hypochlorous acid has been shown to be an effective agent in reducing wound bacterial counts in open wounds. Ultrasound-enabled wound debridement is an effective and efficient method of debridement. This study compared ultrasound irrigation with hypochlorous acid versus saline irrigation for wound debridement on pre- and postoperative wounds and determined regrowth of bacteria over 1 week period of time. Finally, the outcome of definitive wound closure of the clinically clean-appearing wounds was recorded. **Methods:** Seventeen consenting adult patients with chronic open wounds were randomly selected for study. The patients were randomly divided into the hypochlorous acid irrigation or saline irrigation group. All patients provided pre- and postoperative tissue samples for qualitative and quantitative bacteriology. For the time (7 days) between the debridement procedure and the definitive closure procedure, the wounds were dressed with a silver-impregnated dressing and a hydroconductive dressing. **Results**: Both types of irrigation in the ultrasonic system initially lowered the bacterial counts by 4 to 6 logs. However, by the time of definitive closure, the saline-irrigated wounds had bacterial counts back up to 10^5^ whereas the hypochlorous acid–irrigated wounds remained at 10^2^ or fewer. More than 80% of patients in the saline group had postoperative closure failure compared with 25% of patients in the hypochlorous acid group. **Conclusions:** Hypochlorous acid irrigation with ultrasound debridement reduced bacterial growth in chronic open wounds more efficiently than saline alone. Postoperative wound closure outcomes suggest a remarkable reduction in wound complications after wound debridement using hypochlorous acid irrigation with ultrasound versus saline alone.

Wound bed preparation is the management of a wound to accelerate endogenous healing or to facilitate the effectiveness of other therapeutic measures.^1^ The concept of wound bed preparation has evolved to provide a systematic approach to removing the barriers to natural wound healing and enhancing the effects of wound therapies.^2^ To effect wound bed preparation, it is necessary to debride necrotic tissue and debris, decrease excessive wound exudate, decrease the tissue bacterial level, remove deleterious chemical mediators, and set the stage for acceleration of endogenous healing or wound closure by wound approximation, skin graft, or pedicle flap.^3^ This debridement can be accomplished in multiple ways including surgical, mechanical, enzymatic, biological, and autolytic debridement.^4^ In addition to removing necrotic and nonviable tissues, debridement removes tissue laden with bacteria and thus the bioburden.^5^

Low-frequency ultrasonic mechanical debridement is useful in chronic wounds because it can combine mechanical debridement, sharp debridement, and continuous saline irrigation while delivering therapeutic ultrasound to the wound bed.^6^ Although saline is usually used as an irrigant with ultrasonic debridement, no specific irrigation solution has been shown to be universally effective.^7^ In this study, a noncytotoxic wound solution, hypochlorous acid (HA), was investigated versus saline as an irrigation solution with ultrasonic debridement to determine the relative effectiveness at decreasing the tissue bacterial bioburden in chronic wounds and maintaining the wounds in bacterial balance through wound closure.

## METHODS

Seventeen consenting male and female adult patients with chronic stage 3 to 4 wounds of multiple etiologies were randomly selected for study. All patients were stable of metabolic and cardiovascular conditions and without clinical sepsis. All patients received appropriate perioperative antibiotics based on sensitivities and allergic status. All procedures were carried out by a wound surgeon. The patients were alternately divided into 2 groups. Group I patients received HA (Vashe Wound Solution; SteadMed Medical LLC, Fort Worth, Tex) as a wound irrigant with the ultrasonic debridement, and group II patients received saline as an irrigant with the ultrasonic debridement. Debridement was performed in both groups using Misonix low-frequency ultrasound (SonicOne OR, Ultrasonic Debridement System, Misonix Inc, Farmingdale, NY). All patients were anesthetized with general anesthesia or local anesthesia with monitored sedation for surgical procedures, depending on the patient's general condition.

Pre- and postdebridement tissue biopsies were obtained and immediately placed in liquid nitrogen for storage. Following debridement, all wounds were treated with silver-impregnated, time-release, custom-fit dressings, left in place undisturbed for 7 days. Drawtex hydroconductive wound dressings (SteadMed Medical LLC) were used as an outer dressing and held in place with an appropriate size foam dressing. The outer dressings were changed on alternate days or as needed, depending on the volume of wound exudate. All patients remained at bed rest using low-pressure mattresses throughout the study.

At 7 days following the initial debridement, all patients were returned to the operating room, where they were anesthetized, dressings removed, and the wounds biopsied for bacterial quantification. Samples were labeled and stored in liquid nitrogen. An additional biopsy sample was obtained for standard aerobic and anaerobic culture and sensitivities. All patients, following biopsy at 7 days, underwent selective wound closure at that time. The results of surgical closure were recorded at 7 to 10 days following wound closure.

Data were recorded pre- and postoperative debridement on day 1, at 7 days postdebridement prior to wound closure, and 7 to 10 days following wound closure. Clinical outcome data were compared 3 weeks after the definitive surgery. Mean bacterial counts for each patient group were determined at each time point and expressed as CFU (colony-forming unit) per gram of tissue. These values were compared at each time point using a 1-way analysis of variance. Post hoc analyses of differences between groups were carried out using Tukey's test, with *P* < .05 considered significant. Sigma Stat statistical software (Jandel Scientific, Corte Madera, Calif) was used for data analysis.

## RESULTS

**Figure 1:**
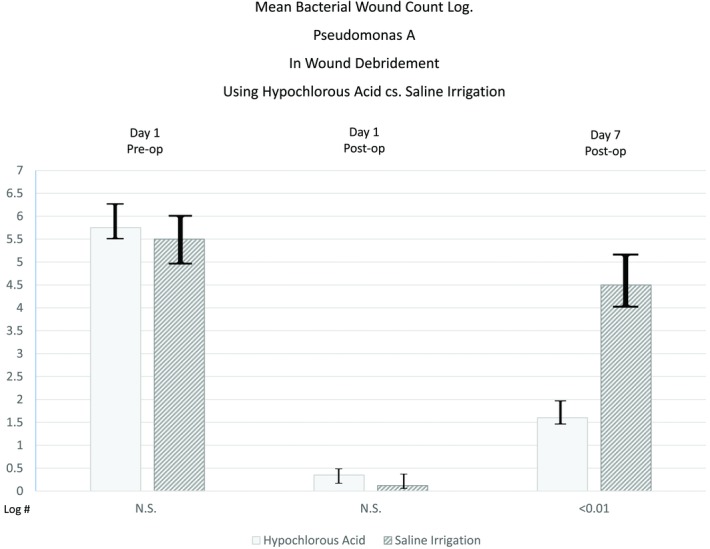
Mean bacterial wound count log. *Pseudomonas aeruginosa* in wound debridement using hypochlorous acid versus saline irrigation.

The results of this clinical study are demonstrated in Tables 1-4 and Figure 1. Tables 1 and 2 reflect the patient demographics and wound type. Tables 3 and 4 demonstrate comparable effective bacterial bioburden reduction in both groups I and II immediately after debridement. At 7 days after debridement, a significant difference (*P* < .05) was observed between the 2 groups. Group I treated with HA irrigation showed sustained suppression of bacterial growth. However, group II patients treated with saline irrigation showed growth of bacteria to near predebridement levels at 7 days.

The clinical outcomes of surgical wound closure demonstrated fewer postoperative complications in group I patients (25%) than in group II patients (>80%). The wounds in both groups were not clinically distinguishable one from the other group at 7 days postdebridement when the wounds were scheduled for closure. These observations were made by 1 experienced surgeon without knowledge of the bacterial quantification results. One patient with complication in group I died 7 days after sacral wound closure due to myocardial disease. The wound closure at that time, however, was intact and did not appear to contribute to the mortality.

## DISCUSSION

To effect adequate debridement, nonviable tissue, debris, and bacteria must be removed from chronic wounds. A high tissue bacterial bioburden has been associated with a failure of wound healing.^6^ The level of tissue bacterial bioburden that inhibits healing has been shown in multiple studies to be greater than 10^5^ or at least 1 × 10^6^ bacteria per gram of tissue.^8^^,^^9^ Such high levels of tissue bacteria can be present without clinical signs of infection and when present can deleteriously affect wound healing^10^ and prevent wound closure by wound approximation, skin graft, or pedicle flap.^8^

Low-energy ultrasonic mechanical debridement as used in this study has been reported to effectively debride wounds without causing excessive tissue trauma.^9^ Advantages of ultrasound to healing include release of nitrous oxide via fluid shear stress stimulation of cells resulting in resolution of vasospasm, thereby increasing blood flow around the wound.^11^ In addition, fibroblasts, macrophages, and endothelial cells are stimulated.^12^

Wound antiseptics have been useful as wound irrigants, but some such as Dakin's solution and chlorhexidine have been reported to be cytotoxic.^13^^,^^14^ Hypochlorous acid has been demonstrated to be noncytotoxic, so was chosen as an irrigant in this study.^15^ The antibacterial effects of HA maintained the debrided wounds in bacterial balance during the 7-day period until wound closure was performed. Despite the fact that clinically the wounds in both groups I and II patients appeared ready for closure, the wounds in group II patients that had been irrigated with saline had an increased bioburden and more than 80% of the wound closures were unsuccessful. This lack of correlation between clinical judgment and bacterial invasion of the tissue was as reported by Serena et al.^10^

## CONCLUSIONS

This study demonstrates that ultrasound debridement is an effective method to lower tissue bacterial counts in chronic wounds. It also demonstrated that HA is more effective than saline as an irrigant with ultrasonic debridement for maintaining wounds post–initial debridement until wound closure can be performed. The results of surgical closure reconfirms that the bacterial load in wounds influences closure outcome when clinical judgment cannot discriminate between wounds with a high bioburden and those in bacterial balance. Using tissue bacterial levels can predict safe wound closure and reduce the need for repeated debridement or staging of wound closure. Finally, the clinical outcomes of surgical wound closure of chronic wounds 7 days after ultrasonic debridement were superior when HA was used as an irrigant in the ultrasonic debrider as compared with the more commonly used saline as an irrigant.

## Figures and Tables

**Table 1:** Group I hypochlorous acid irrigation

Patient demographics				Wound characteristics		
Patient	Ethnic background	Sex	Age	Wound type	Osteomyelitis	Wound location
1_I_	African American	♂	21	Pressure sore Stage IV Paraplegic	+	L. ischium
2_I_	Caucasian	♀	49	Pressure sore Stage IV	+	Sacrum
3_I_	Caucasian	♀	69	Pressure sore Stage IV Multiple sclerosis	+	L. ischium
4	Caucasian	♂	32	Pressure sore Stage IV paraplegic	+	L. ischium
5_I_	Caucasian	♂	43	Venous diabetic ulcers	O	L. leg
6_I_	African American	♀	56	Venous ulcer Stage III	O	L. leg
7_I_	Caucasian	♀	61	AK Stump 2-y infection Obese Diabetic	O	R. thigh
8_I_	Caucasian	♀	58	Multiple pressure sores Stage IV x 3	+	Sacrum L. and R. ischium
9_I_	Caucasian	♀	49	Pressure sore Stage IV	O	Sacrum L. ischium

**Table 2:** Group II saline irrigation in ultrasound debridement

**Patient demographics**				**Wound characteristics**		
Patient	Ethnic background	Sex	Age	Wound type	Osteomyelitis	Wound location
1_II_	Hispanic	♂	32	Pressure sore Stage IV Paraplegic	+	L. ischium
2_II_	African American	♂	21	Pressure sore Stage IV Paraplegic	+	L. ischium
3_II_	Caucasian	♀	56	Pressure sore Stage IV Obese	+	Sacrum
4_II_	Caucasian	♀	54	Pressure sore Stage IV Paraplegic Obese	O	Sacrum
5_II_	Caucasian	♂	43	Venous/traumatic ulcer Stage III Obese	O	L. lower leg
6_II_	Caucasian	♀	65	Pressure sore Stage III Obese	+	L. buttock
7_II_	Caucasian	♂	46	Pressure sore Stage IV Obese	O	Sacrum
8_II_	Caucasian	♂	62	Diabetic ulcer Stage III	O	R. foot

**Table 3:** Group I hypochlorous acid irrigation: Wound bacterial quantification and surgical closure outcome

Patient	Day 1 preoperative debridement bacterial count	Day 1 postoperative debridement bacterial count	Day 7 preoperative closure bacterial count	Day 14 wound closure outcome
1_I_	X *Pseudomonas* 10^6^ K *Klebsiella* 10^4^	X: 10^2^ K: 10^2^	X: 10^2^ K: 10	Wound healed No complications
2_I_	X *Pseudomonas* 10^7^ S *Staphylococcus aureus* 10^5^ E *Enterococcus* 10^6^	X: 10^3^ S: 0 E: 10^2^	X: 10 S: 0 E: 10^2^	Wound healed No complications
3_I_	X *Pseudomonas* 10^6^ S *Staph*ylococcus *aureus* 10^5^	X: 0 S: 10^2^	X: 10^2^ S: 10^2^	Wound healed No complications
4_I_	X *Pseudomonas* 10^7^ S *Staphylococcus aureus* 10^4^	X: 0 S: 0	X: 10^2^ S: 0	Wound healed No complications
5_I_	X *Pseudomonas* 10^5^ S *Staph*ylococcus aureus 10^5^	X: 10 S: 0	X: 10 S: 0	
6_I_	X *Pseudomonas* 10^5^ E *Escherichia coli* 10^5^	X: 10 E: 0	X: 10^2^ E: 0	Wound healed
7_I_	X *Pseudomonas* 10^4^ S *Staphylococcus aureus* 10^5^ E *Enterococcus* 10^4^	X: 10 S: 0 E: 10	X: 10^2^ S: 10 E: 0	2-cm wound Dehiscence 2^o^ to delayed anticoagulation
8_I_	X *Pseudomonas* 10^4^ S *Staphylococcus aureus* 10^5^	X: 0 S: 0	X: 10 S: 10	Day 6 100% Graft
9_I_	X *Pseudomonas* 10^5^ K *Klebsiella* 10^4^	X: 10 K: 10	X: 10^2^ K: 0	Small partial dehiscence

**Table 4:** Group II saline irrigation: Wound bacterial quantification and surgical closure outcome

Patient	Day 1 preoperative debridement	Day 1 postoperative debridement	Day 7 preoperative closure	Day 14 wound closure outcome
1_II_	X *Pseudomonas* 10^6^ S *Staphylococcus aureus* 10^3^	X *Pseudomonas* 10^2^ S *Staphylococcus aureus* 0	X *Pseudomonas* 10^5^ S *Staphylococcus aureus* 10^5^	Wound dehiscence Day 4 postoperatively
2_II_	X *Pseudomonas* 10^7^ E *Enterococcus* 10^4^	X *Pseudomonas* 10 E *Enterococcus* 0	X *Pseudomonas* 10^2^ E *Enterococcus* 10^2^	Wound closed No complication
3_II_	X *Pseudomonas* 10^6^ S *Staphylococcus aureus* 10^5^	X *Pseudomonas* 10^2^ S *Staphylococcus aureus* 0	X *Pseudomonas* 10^4^ S *Staphylococcus aureus* 10^4^	Wound closed Small wound dehiscence
4_II_	X *Pseudomonas* 10^5^ S *Staphylococcus aureus* 10^6^ E *Enterococcus* 10^4^	X *Pseudomonas* 0 S *Staphylococcus aureus* 10^2^ E *Enterococcus* 0	X *Pseudomonas* 10^3^ S *Staphylococcus aureus* 10^4^ E *Enterococcus* 10^5^	Wound closed Postop infection Day 4
5_II_	X *Pseudomonas* 10^4^ S *Staphylococcus aureus* 10^4^	X *Pseudomonas* 10 S *Staphylococcus aureus* 0	X *Pseudomonas* 10^5^ S *Staphylococcus aureus* 10^3^	Wound closed Postoperative infection Graft lost
6_II_	S *Staphylococcus aureus* 10^5^	S *Staphylococcus aureus* 10	S *Staphylococcus aureus* 10^5^	Graft failed
7_II_	X *Pseudomonas* 10^6^ S *Staphylococcus aureus* 10^5^	X *Pseudomonas* 10 S *Staphylococcus aureus* 10	X *Pseudomonas* 10^4^ S *Staphylococcus aureus* 10^5^	Partial dehiscence Day 5
8_II_	X *Pseudomonas* 10^6^ S *Staphylococcus aureus* 10^4^ E *Escherichia coli* 10^5^	X *Pseudomonas* 10^2^ S *Staphylococcus aureus* 0 E *Escherichia coli* 10	X *Pseudomonas* 10^5^ S *Staphylococcus aureus* 10^2^ E *Escherichia coli* 10^5^	Wound infection Day 4
